# Membrane Fatty Acid Composition and Cell Surface Hydrophobicity of Marine Hydrocarbonoclastic *Alcanivorax borkumensis* SK2 Grown on Diesel, Biodiesel and Rapeseed Oil as Carbon Sources

**DOI:** 10.3390/molecules23061432

**Published:** 2018-06-13

**Authors:** Maria Konieczna, Martin Olzog, Daniela J. Naether, Łukasz Chrzanowski, Hermann J. Heipieper

**Affiliations:** 1 Department of Environmental Biotechnology, Helmholtz Centre for Environmental Research-UFZ, Permoserstr, 15, 04318 Leipzig, Germany; maria.konieczna@ufz.de (M.K.); Martin-Olzog@web.de (M.O.); 2 Department of Molecular Biosciences, Goethe University Frankfurt, Max-von-Laue-Str, 9, 60438 Frankfurt, Germany; daniela.naether@gmx.de; 3 Faculty of Chemical Technology, Poznan University of Technology, 60-965 Poznan, Poland; lukasz.chrzanowski@put.poznan.pl

**Keywords:** *Alcanivorax borkumensis* SK2, biodiesel, cell surface hydrophobicity, degree of saturation, diesel, growth rates, marine oil spills, membrane fatty acids, *trans/cis* ratio, water contact angles

## Abstract

The marine hydrocarbonoclastic bacterium *Alcanivorax borkumensis* is well known for its ability to successfully degrade various mixtures of *n*-alkanes occurring in marine oil spills. For effective growth on these compounds, the bacteria possess the unique capability not only to incorporate but also to modify fatty intermediates derived from the alkane degradation pathway. High efficiency of both these processes provides better competitiveness for a single bacteria species among hydrocarbon degraders. To examine the efficiency of *A. borkumensis* to cope with different sources of fatty acid intermediates, we studied the growth rates and membrane fatty acid patterns of this bacterium cultivated on diesel, biodiesel and rapeseed oil as carbon and energy source. Obtained results revealed significant differences in both parameters depending on growth substrate. Highest growth rates were observed with biodiesel, while growth rates on rapeseed oil and diesel were lower than on the standard reference compound (hexadecane). The most remarkable observation is that cells grown on rapeseed oil, biodiesel, and diesel showed significant amounts of the two polyunsaturated fatty acids linoleic acid and linolenic acid in their membrane. By direct incorporation of these external fatty acids, the bacteria save energy allowing them to degrade those pollutants in a more efficient way. Such fast adaptation may increase resilience of *A. borkumensis* and allow them to strive and maintain populations in more complex hydrocarbon degrading microbial communities.

## 1. Introduction

Marine hydrocarbonoclastic bacteria constitute a very specialized group of oil-degrading γ-proteobacteria that was intensely studied during the last decade as one of the key players for biodegradation of marine crude oil contaminations [1,2,3]. These bacteria are only able to metabolize a few organic acids (acetate, pyruvate), and feed exclusively on a variety of aliphatic hydrocarbons [1]. Cells of this very important group of marine bacteria, which has *Oleispira* and *Alcanivorax* as the best investigated genera, have been discovered occurring in very small abundances in most of the seas all over the world [4].

*Alcanivorax borkumensis* SK2 as the most studied marine hydrocarbonoclastic bacterium can only use different aliphatic hydrocarbons and pyruvate as sole carbon and energy source [4]. Due to the ability of marine hydrocarbonoclastic bacteria to utilize a broad variety of aliphatic hydrocarbons, they are responsible for microbial blooming during oil spills and can represent up to 80–90% of the bacterial community [5,6]. The genome *A. borkumensis* was sequenced [3] and proteome studies have also been performed [7]. The genome encodes different metabolic ways to catalyze the initial oxidation step of alkane degradation to the corresponding *n*-alkanol intermediates which are very common among proteobacteria [3,5,8].

Recent investigations on growth behavior and physiology of *A. borkumensis* cultivated on n-alkanes with different chain lengths (C6–C30) as substrates revealed increasing growth rates with increasing alkane chain length up to a maximum between C12 and C19 [9]. *A. borkumensis* was found to incorporate and modify fatty acid intermediates generated by the corresponding *n*-alkane degradation pathway [9].

In this contribution, we examined growth dynamics, membrane composition and cell surface hydrophobicity of *A. borkumensis* SK2 cultivated on diesel, biodiesel or rapeseed oil as sole carbon and energy source, in comparison with pyruvate and hexadecane as standard substrates.

## 2. Results

### 2.1. Effect of Growth Substrate on Growth Rates and Cell Surface Properties of A. Borkumensis

**Figure 1:**
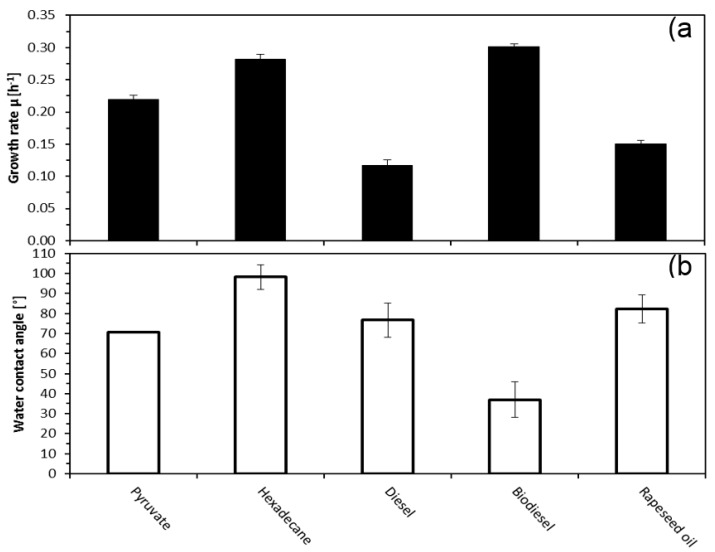
Effect of different growth substrates on growth rates (**a**) and water contact angles (**b**) of *A. borkumensis*.

*Alcanivorax borkumensis* SK2 was cultivated in ONR7 mineral medium using either diesel, biodiesel or rapeseed oil as sole carbon and energy source. In addition, pyruvate (the only known non-alkane growth substrate for this bacterium) and hexadecane (standard *n*-alkane) served as reference substrates. The bacteria were cultivated in water shake batch cultures with 0.5% (*v*/*v*) of corresponding substrates. Exponential phase growth rates µ (h^−1^) for each substrate were calculated according to [10] and are presented in Figure 1a.

The absolute growth rates µ (h^−1^) and corresponding doubling times t_D_ (h) were the highest with biodiesel (µ: 0.302 h^−1^, t_D_: 2.3 h) and hexadecane (µ: 0.282 h^−1^, t_D_: 2.5 h). Pyruvate (µ: 0.220 h^−1^, t_D_: 3.2 h). Rapeseed oil and diesel grown cells corresponded to significantly smaller growth parameters (rapeseed oil µ: 0.15 h^−1^, t_D_: 4.6 h; diesel µ: 0.117 h^−1^, t_D_: 5.9 h). General tendency was that growth on biodiesel was very similar to growth on alkanes and pyruvate, whereas growth with rapeseed oil and diesel was lower.

To investigate the effect of different carbon and energy sources on the cell surface hydrophobicity, the water contact angles were determined (Figure 1b). Water contact angles (θ_w_) of cells grown with hexadecane showed the highest values with 98°, followed by rapeseed oil (θ_w_ 82°), diesel (θ_w_ 76°), and pyruvate (θ_w_ 70°). Contrary to those highly hydrophobic cell surfaces, cells grown, cell grown with biodiesel showed quite hydrophilic surface properties with water contact angles of (θ_w_ 37°).

### 2.2. Effect of Growth Substrate on Membrane Fatty Acid Composition of A. Borkumensis

As shown previously [9,11], typical membrane of *A. borkumensis* SK2 grown on pyruvate or *n*-hexadecane, respectively, consists of the following fatty acids: C14:0, C16:0, C16:1Δ9*trans*, C16:1Δ9*cis*, C18:0, C18:1Δ11*trans*, and C18:1Δ11*cis*.

**Figure 2:**
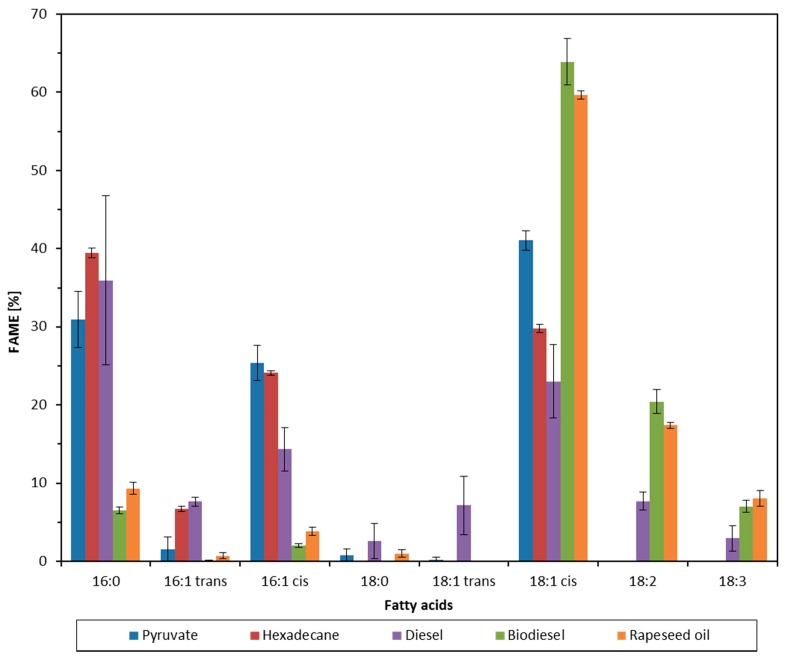
Fatty acid patterns of *A. borkumensis* grown with different substrates as sole carbon and energy source.

When comparing fatty acid patterns of cells grown on all substrates tested (Figure 2) significant differences in the relative abundances of certain fatty acids can be observed. The most obvious variation is related to the presence of 18:1*cis* in cells grown with biodiesel and rapeseed oil. Cells grown on rapeseed oil and biodiesel showed a far higher amount of 18:1*cis* in their membranes. In addition, cells grown on rapeseed oil, biodiesel and diesel showed considerable amounts of the two polyunsaturated fatty acids linoleic acid (C18:2Δ9,12*cis,cis*) and linolenic acid (C18:3Δ9,12,15*cis,cis,cis*) in their membrane fatty acids. It should be emphasized that these fatty acids cannot be synthesized by the bacteria and must have been directly incorporated from these substrates.

Since 2009, in Germany and other EU countries, an average of 7% (*v*/*v*) biodiesel is added to regular diesel fuel (the so-called B7 diesel). This also explains why 18:2 and 18:3 fatty acids were detected in the membrane of cells grown with diesel.

Using the fatty acid patterns of cells grown with different substrates, two sum parameters regularly used to express the overall rigidity and membrane stress responses were calculated.

**Figure 3:**
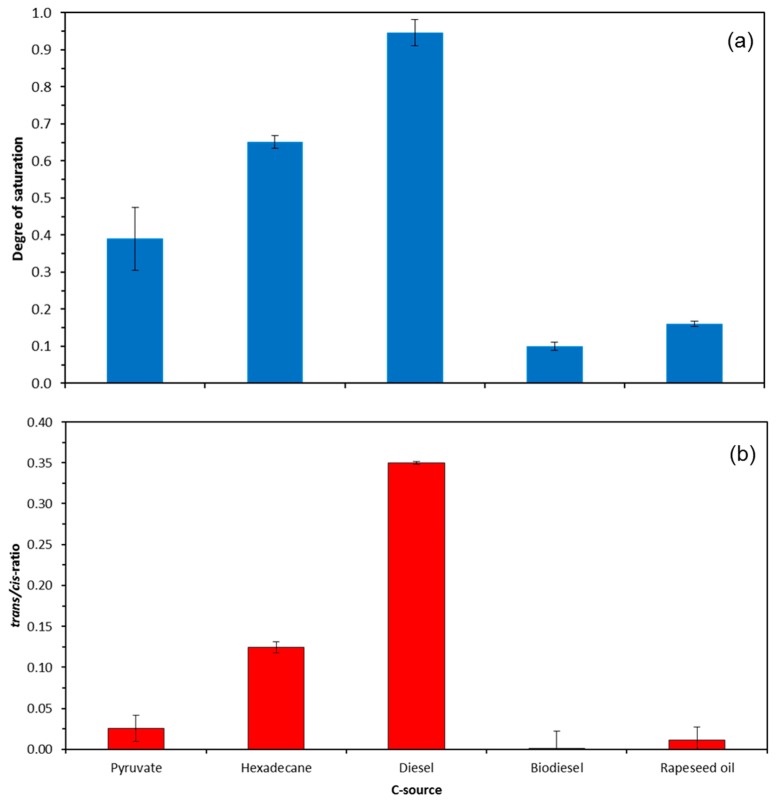
Effect of different growth substrates on degree of saturation (**a**) and *trans/cis* ratio of unsaturated fatty acids (**b**) of *A. borkumensis*.

Both the degree of saturation and the *trans/cis* ratio of unsaturated fatty acids were by far the highest in cells grown with diesel as sole carbon and energy source followed by hexadecane. Contrary to that, both sum parameters showed only very low values for cells grown with pyruvate, rapeseed oil, and biodiesel (Figure 3).

## 3. Discussion

Bacterial bioremediation of marine oil spills is still present in public awareness due to the ongoing discussions about compensations and responsibilities connected to the environmental catastrophe of the *Deepwater Horizon* oil spill in the Gulf of Mexico in 2010 [12,13,14]. Here and in other marine environments polluted by crude oil, marine hydrocarbonoclastic bacteria such as *A. borkumensis* were detected as key players in bioremediation [15,16,17,18]. *A. borkumensis* was therefore already intensively studied regarding to genetics and metabolic adaptation [2,3,7]. However, detailed investigations on growth behavior and adaptive responses of these bacteria to other substrates were still missing. This is especially important in terms of *A. borkumensis* efficiency to overgrow different species during initial microbial blooming. This might especially occur because of the high ability of the bacteria to both modify and incorporate fatty intermediates derived from the alkane degradation pathway to save energy while growing on alkane-like substrates. The highest growth rates could be shown for *A. borkumensis* cultivated on biodiesel, which therefore represents a substrate similar to the hexadecane (standard n-alkane substrate) as well as to the pyruvate (second standard substrate). Contrary to that, *A. borkumensis* grown on rapeseed oil and diesel showed significantly lower growth rates. Since biodiesel in central Europe is mainly produced from rapeseed oil, similar growth rates would have been expected. However, rapeseed oil consists of triglycerides, where three fatty acids of chain length C14–C18 are esterified to one molecule of glycerol. During production of biodiesel, the triglycerides are methylated by transesterification to fatty acid methyl esters (FAME) to the so-called rapeseed methyl esters form. Rapeseed oil consists of 66.6% 18:1Δ9*cis*, 18.7% 18:2, and 7.7% 18:3 [19]. Despite this similarity in the composition of the substrate, only very low growth rates could be observed for *Alcanivorax* cultivated on rapeseed oil compared to biodiesel. Moreover, compared to rapeseed oil, biodiesel is usually protected from oxidation by the addition of tert-butylhydroquinone which can hinder biodegradation [20]. However, in our experiments no negative influence was observed. Rapeseed oil can be also protected by antioxidants however the mode of their action would be different as they should eliminate oxidation of oil in higher temperature and are all food-safe additives. Additionally, active concentration of antioxidants would be very low as *A. borkumensis* was cultivated on 0.5% carbon source. Therefore, negative effects from antioxidants can be omitted.

The lower affinity of *A. borkumensis* towards rapeseed oil in comparison to biodiesel seems to be astonishing. This bacterium is commonly reported to be a highly specialized and restricted to alkanes as the sole carbon source. Alkanes after initial oxidation to corresponding *n*-alkanols, are then further transformed into appropriate fatty acids. Thus, theoretically, there should be no difference between biodiesel (methyl esters of rapeseed oil fatty acids) and untreated rapeseed oil (triglycerides). Our results proved that *A. borkumensis* was either unable to take up the triglycerides of rapeseed oil or to hydrolyze the ester bonds of the triglycerides. Furthermore, the hydrophobicity of the substrate seems to play a role, since the lipophilicity of the cell surface shows an adaptation. Similar to rapeseed oil, diesel showed only low growth rates, even though this substrate consists to a great extent from C9–C26 alkanes, to which 7% biodiesel is blended according to the current requirements of the diesel fuel standard DIN 51268 [21]. However, next to alkanes and cycloalkanes, diesel also accounts for up to 25% of aromatic hydrocarbons with monoaromatic (BTEX) and polyaromatic hydrocarbons (PAH) as main groups of compounds. Especially the BTEX compounds inhibit microbial growth in excessively high concentrations; this might explain the low growth rates on diesel. The addition of biocides to diesel fuel can be neglected as those compounds are active only at the initial step of microbial colonization when small amount of water starts to form a second phase. Further evidence of the toxicity of diesel as a substrate provides both the significantly higher degree of saturation and the *trans/cis* ratio of unsaturated fatty acids [22,23,24].

Various alkane-degrading bacteria can directly incorporate the fatty acids intermediates of the alkane degradation directly into their membrane phospholipids. For *Alcanivorax*, the spectrum of directly incorporated fatty acids lies between fatty acids with 13 and 18 carbon atoms. Using enzymes such as desaturases, elongases and the *cis-trans* isomerase, the corresponding fatty acids are modified [9]. During growth on pyruvate and hexadecane, the fatty acid profile of *A. borkumensis* consists of the saturated fatty acids 16:0 and 18:0, and the unsaturated fatty acids 16:1Δ9*cis*, 16:1Δ9*trans*, 18:1Δ11*cis* and 18:1Δ11*trans* [9,11], and could also be shown in the present work. Cultures cultivated on hexadecane and diesel showed the highest levels of palmitic acid (16:0) closely followed by this obtained on pyruvate. Since diesel also contains alkanes shorter than C16, the corresponding fatty acids with fewer than 16 carbon atoms are elongated by the means of elongases by C2 units [9]. The fact that cultures on rapeseed oil and biodiesel had significantly lower palmitic acid concentrations corresponds to the palmitic acid content in rapeseed oil and biodiesel, which is around 5%. It can be concluded that *Alcanivorax* grown on rapeseed oil synthesizes palmitic acid de novo, which may also apply due to its similarity to biodiesel. Similarly, the composition of rapeseed oil and biodiesel, are found in the fatty acid schemes mainly oleic acid (18:1Δ9*cis*), followed by linoleic acid (18:2) and linolenic acid (18:3). Linoleic acid and linolenic acid were also observed in diesel cultures that contains also 7% of biodiesel. It can be assumed that these fatty acids were integrated directly into the membrane, since both the cultures on pyruvate and on hexadecane showed no polyunsaturated fatty acids, which was also shown in previous work [9]. The presence of linoleic and linolenic acid within the membrane lipids is unusual for cultures grown on diesel. This can, however, be explained by the addition of 7% biodiesel to diesel fuel according to the diesel fuel standard DIN 51268. The ratios of saturated to unsaturated fatty acids are a measure of the adaptation of bacteria to stress at the level of the membrane [23,24,25]. The fact that the degree of saturation with diesel as substrate was significantly higher than for all other substrates tested is an important indicator of the toxic effect of diesel on *A. borkumensis*. Moreover, the calculated *trans/cis* ratios of unsaturated fatty acids as an indicator of the activity of *cis-trans* isomerase serve as an important mechanism of adaptation of these bacteria [9,22,25] and reflected these results.

It is a common strategy of several bacteria to change their surface to be more hydrophobic to access hydrophobic substrates [26,27]. This adaptation is also important because bacterial *n*-alkane oxygenase systems are in the membrane and need direct contact with their substrate [28,29,30]. *A. borkumensis*, similar to various oil-degrading bacteria [31], produces a biosurfactant in order to achieve the effect of an increased surface hydrophobicity [1].

It was previously shown that *A. borkumensis* incorporates the acidic degradation intermediates of *n*-alkane into its membrane phospholipids [9]. The membrane accounts for about 10% of the bacterial biomass, and de novo synthesis of fatty acids is very energy consuming. Therefore, bacteria directly incorporating fatty acids into their membranes will be more advantageous compared to those synthesizing fatty acids de novo*.* Only fatty acids with a chain length of C14 to C18 are expected to be incorporated into the membrane and palmitic acid (C16:0) is the dominant fatty acids in *A. borkumensis* and many other bacteria [11]. Hence, it was expected that growth on *n*-hexadecane is faster than growth on pyruvate and actually also faster than on all other supplied substrates. This was the case for similar experiments made with *Rhodococcus erythropolis* [26].

So far, membrane fatty acid composition of *A. borkumensis* was studied only after growth on pyruvate or *n*-hexadecane [11]. As discovered there and also seen in the result presented here, the membrane fatty acid pattern of *A. borkumensis* normally consists of seven fatty acids (14:0; 16:0; 16:1Δ9*cis*; 16:1Δ9*trans*; 18:0; 18:1Δ11*cis*; 18:1Δ11*trans*).

Therefore, the most exciting discovery of this work was the fact that the poly-unsaturated fatty acids 18:2 and 18:3 were found in bacteria grown on diesel, biodiesel, and rapeseed oil. The occurrence of these fatty acids revealed the great ability of this bacterium to incorporate external sources into the membrane fatty acids.

The activity of the active *cis*-*trans* isomerase of unsaturated fatty acids as an urgent mechanism of membrane modification, is another proof that *A. borkumensis* has great capabilities to cope with environmental stress conditions [9,22].

All these adaptations and abilities to assimilate several potential organic pollutants explain why *A. borkumensis* is so important for the bioremediation of oil-polluted marine environments [32].

## 4. Materials and Methods

### 4.1. Strain and Chemicals

*Alcanivorax borkumensis* SK2 (NCIMB 11132) is the reference strain for hydrocarbonoclastic bacteria and has previously been described [11]. All chemicals were reagent grade and obtained from commercial suppliers. Diesel and biodiesel fuel were obtained from a tank station (Leipzig, Germany), rapeseed oil was obtained from a local supermarket (Leipzig, Germany).

### 4.2. Culture Conditions

*A. borkumensis* SK2 (NCIMB 11132) was cultivated at 30 °C in a modified ONR7-medium which contains (per L): NaCl (22.79 g), Na_2_SO_4_ (3.98 g), KCl (0.72 g), NaBr (83 mg), NaHCO_3_ (31 mg), H_3_BO_3_ (27 mg), TAPSO (1.3 g). MgCl_2_·6H_2_O (11.18 g), CaCl_2_·2H_2_O (1.46 g), SrCl_2_·6H_2_O (24 mg). FeCl_2_·4H_2_O (40 mg). NH_4_Cl (5.4 g), Na_2_HPO_4_·7H_2_O (1.78 g), Na_2_HPO_4_ (0.94 g). Trace elements were added from a 500-times concentrated stock solution to final concentrations (per L) MgSO_4_·7H_2_O (100 mg), FeSO_4_·7H_2_O (10 mg), MnSO_4_·H_2_O (5 mg), ZnCl_2_ (6.4 mg), CaCl_2_·6H_2_O (1 mg), BaCl_2_ (0.6 mg), CuSO_4_·7H_2_O (0.36 mg), CuSO_4_·5H_2_O (0.36 mg), H_3_BO_3_ (6.5 mg), EDTA (10 mg). *n*-alkanes (0.5% *v*/*v*) or pyruvate (20 g) were added as carbon and energy source. The pH was set to 7.6 with NaOH. Cells were grown in 100 mL shake cultures in a shaking water bath (Shaker GFL, Burgwedel, Germany) at 200 rpm.

### 4.3. Lipid Extraction, Transesterification, and Fatty Acid Analysis

Membrane lipids were extracted with chloroform/methanol/water as described by Bligh and Dyer [33]. Fatty acid methyl esters (FAME) were prepared by incubation for 15 min at 95 °C in boron trifluoride/methanol applying the method of Morrison and Smith [34]. FAME were extracted with hexane.

### 4.4. Analysis of Fatty Acid Composition by GC-MS

Analysis of FAME in hexane was performed by means of gas chromatography-mass spectrometry (Agilent GC7890A and MS5975C, Boeblingen, Germany) with the following parameters: MS-source at 230 °C, MS-quadrupole at 150 °C, inlet with splitless mode at 280 °C, HP-5MS column (30 m × 0.25 mm × 0.25 µm) at 50 °C for 1 min then 4 °C min^−1^ to 250 °C, 20 °C min^−1^ to 300 °C for 5 min and finally a 10 min post-run at 300 °C. The mass spectra were evaluated using a library of previously determined mass spectra of fatty acids standards.

### 4.5. Analysis of Fatty Acid Composition by GC-FID

Analysis of FAME in hexane was performed using a quadruple GC System (HP5890, Hewlett & Packard, Palo Alto, CA, USA) equipped with a split/splitless injector and a flame ionization detector (FID). A CP-Sil 88 capillary column (Chrompack, Middelburg, The Netherlands; length, 50 m; inner diameter, 0.25 mm; 0.25 µm film) was used for the separation of the FAME. GC conditions were: Injector temperature was held at 240 °C, detector temperature was held at 270 °C. The injection was splitless, carrier gas was He at a flow rate of 2 mL min^−1^. The temperature program was: 40 °C, 2 min isothermal; 8 °C min^−1^ to 220 °C; 15 min isothermal at 220 °C. The peak areas of the FAMEs were used to determine their relative amounts. The fatty acids were identified by GC and co-injection of authentic reference compounds obtained from Supelco (Bellefonte, PA, USA). The degree of saturation of the membrane fatty acids was defined as the ratio between the saturated fatty acids and the unsaturated fatty acids present in these bacteria.

### 4.6. Characterisation of Bacterial Cell Surface Hydrophobicities

Physico-chemical cell surface properties of bacteria were investigated using standard methods as described by others [35]. Bacterial lawns needed for contact angle (*θ*_w_) measurements were prepared by collecting cell suspensions in 10 mM KNO_3_ on 0.45-μm pore-size Micropore filters (Schleicher & Schuell, Dassel, Germany), mounting the filters on glass slides, and drying them for 2 h at room temperature. Cells exposed to the solvents were washed 6 times with 10 mM KNO_3_ to guarantee the measurement of the cell properties and concomitantly to avoid measurement interferences by the physico-chemical effect of the solvents adhering to or accumulating in the cells. Cell surface hydrophobicities were derived from *θ*_w_ of water drops on the bacterial lawns using a Krüss drop shape analysis system DSA 100 (Krüss GmbH, Hamburg, Germany) [35]. According to an earlier classification, cells exhibiting contact angles of *θ*_w_ < 20°, 20° ≤ *θ*_w_ ≤ 50° and *θ*_w_ > 50° are hydrophilic, intermediately hydrophilic and hydrophobic, respectively [36].

### 4.7. Statistical Analysis

All experiments were carried out in triplicates. The obtained data were analyzed via analysis of variance (ANOVA) to detect significant differences between the data. The probability of α (type I error) was 5% (*p* < 0.05).
